# Meat Consumption, Sustainability and Alternatives: An Overview of Motives and Barriers

**DOI:** 10.3390/foods12112144

**Published:** 2023-05-26

**Authors:** Maria Font-i-Furnols

**Affiliations:** IRTA-Food Quality and Technology, Finca Camps i Armet, 17121 Monells, Girona, Spain; maria.font@irta.cat

**Keywords:** sustainable meat, organic meat, edible offal, algae, fungi, insect, plant-based, cultured ‘meat’, motives, barriers

## Abstract

Meat and meat products are important sources of protein in the human diet. However, their consumption or excessive consumption has been questioned as this has been related to sustainability and health issues. Due to this, alternatives to conventional meat consumption, such as meat produced more sustainably or meat alternatives, have been considered. The aim of the present work is to gain insight into the meat consumption of different countries, the motives for and barriers to this consumption, as well as into the consumption of more sustainably produced meat with particular focus on organic meat and meat alternatives. Information on meat consumption has been obtained using FAOSTAT data and maps have been constructed using SAS software. Results showed that, in general, albeit with variations between and within countries, there is a tendency to decrease red meat consumption and increase poultry consumption, while for pork consumption the tendency is less clear. Motives and barriers for meat and meat alternative consumption have been reviewed and it is possible to see that these are very variable and that they, in addition to the intrinsic and extrinsic characteristics of the meat, are also related to consumers’ attitudes and beliefs. Thus, it is important to inform consumers in a truthful and reliable way in order to allow them to make well-founded decisions regarding the consumption of these products.

## 1. Introduction

Meat is an important food and a major source of protein in the diet of many people, and humans are adapted to eat meat. In addition, meat is a significant source of nutrients such as vitamins and minerals, some of which are only available, or have greater bioavailability, in animal products [1,2]. However, in the last decades meat production and consumption have been questioned due to a number of issues. In this regard, a major concern for the livestock and meat sector relates to the sustainability and ethical aspects of meat production, together with the health issues related to meat consumption.

According to the Organization for Economic Co-Operation and Development (OECD)/Food and Agriculture Organization of the United Nations (FAO) [3], the meat production forecast for 2030 indicates that there will be a global increase. Poultry meat will continue to have the greatest growth since, due to its short production cycle, it would be quicker to achieve improvements in its production. The highest expansion is foreseen in Asia. With regards to pork, its production is expected to increase in China and Vietnam, after the outbreak of Asian Porcine Fever, and also in Russia. However, in the EU, due to environmental concerns, it is predicted to decrease until 2030. Beef production is expected to grow mainly in Sub-Saharan Africa and, to a lesser extent, in America, while it is expected to decrease in Europe and principally in India. With respect to lamb production, the highest increase is predicted in Africa and a more moderate increase in Oceania [3]. However, it is possible that production systems worldwide could suffer reversals due to input costs in production, animal diseases, climate change or pandemic effects [4]. 

On the other hand, according to the OECD/FAO [3], meat consumption is expected to increase by 14% until 2030, mainly due to the increase in population (expected to be at 11%). This increase in meat consumption is predicted to be higher in Africa (30%), followed by Asia and the Pacific Region (18%), Latin America (12%), North America (9%) and much less in Europe (0.4%). The highest increase will be of poultry meat, which will have an important role in the diets of populations mainly from developing countries, China and India included. This will be followed by pork, which is expected to increase in Latin America and in some Asian countries, but is expected to decrease in Europe. Regarding beef, a decrease is foreseen by 2030, decreasing in America, Europe, Australia and New Zealand and only increasing in some Asian countries [3]. When studying a longer period of time, up to 2050, it is expected that meat consumption will increase by 17% globally if the historical trend continues without change, 21% if all the current challenges to improve production systems are not met, or by only 3% if action is taken to become more sustainable and equitable. In the last case, in European and American regions, meat consumption would decrease while increasing in the other regions of the world [5].

Thus, it is important to highlight that, according to the United Nations’ Sustainable Development Goal (SDG) 12, there is a need to change patterns of food production and consumption in order to make them more sustainable, considering aspects of the three planetary crises: climate change, biodiversity loss and pollution [6]. Climate change is already perceptible through increased heat waves, forest fires, floods and droughts, and it affects arable and livestock food production, which must adapt to the changes in order to be resilient and efficient so as to provide sufficient food for the world’s populations. Strategies must be developed to be more sustainable, with greater efficiency, producing fewer residues and less contamination and for driving a circular economy [6]. In this sense, and regarding meat production, the livestock and meat processing sectors are working to decrease their environmental impact, increase animal welfare, food security and safety in order match the societal demands [7]. Furthermore, food industries are also working to provide meat alternatives with the aim of partially or completely replacing meat. 

With regard to meat consumption, it is important to understand the existing trends in different countries and regions in order to be aware of differences or possible patterns, as well as to understand the reasons for these. This would provide an overview of the current situation and provide insight into what may happen in the future and how this could be handled. Moreover, to overcome possible problems due to unsustainable meat production and excessive meat consumption, options could include the production and consumption of more sustainable meat and/or meat alternatives, and therefore it would be of great importance to be aware of the possible reasons or obstacles to this.

Therefore, the present work aims to gain an insight into meat consumption in different countries, the reasons for and barriers to this consumption, as well as into the consumption of more sustainably produced meat, with particular focus on organic meat and meat alternatives. 

## 2. Meat Consumption

There are several works in the literature that report on the consumption of meat, but these concern specific countries and areas and it is difficult to find information comparing consumption from all countries. Due to this, in order to have a general overall view of the consumption of these products, this section includes the consumption of several types of meat by country according to FAOSTAT [8]: red meat (including bovine, mutton and goat meat), pork and poultry. The maps were made using the GMAPS procedure from SAS Software (SAS Institute Inc., Cary, NY, USA). In each section, the consumption in 2020 and the difference in consumption over the last ten years (between 2020 and 2010) is presented for the different types of meat. Due to the pandemic situation in 2020, some trends in meat consumption may have changed. Thus, meat consumption in 2019 and the difference between 2019 and 2010 were also evaluated. The results are not presented, but the most relevant changes will be noted.

### 2.1. Red Meat Consumption 

**Figure 1:**
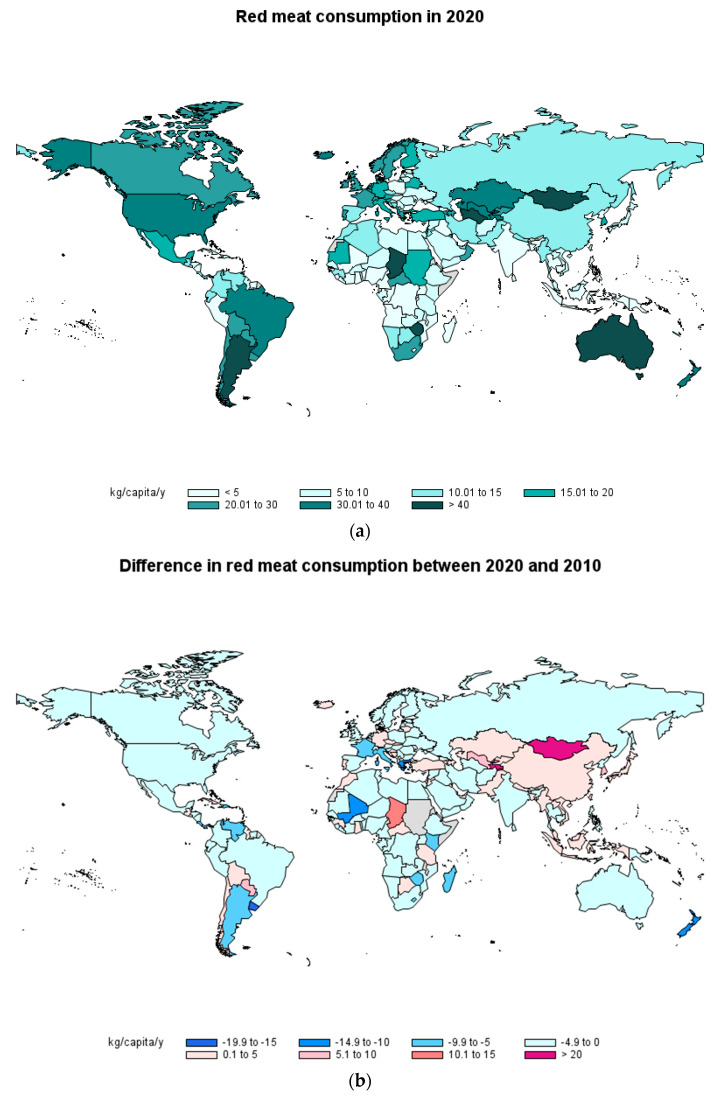
Consumption of red meat in 2020 by country (**a**) and difference in consumption of red meat between 2020 and 2010 (**b**) in kg/capita/year (Source: FAOSTAT [8]).

Consumption of red meat in 2020 [8] varied noticeably by country, as can be seen in Figure 1a. A quick overview of the map allows high-consumption regions such as North America and most of South America, Europe, Oceania and the central part of Asia to be identified. Important differences were observed between African countries. The highest values were for Mongolia (92.89 kg/capita/y, being 71.4% mutton and goat consumption), followed by Argentina (48.35 kg/capita/y, 97.1% of this consumption being bovine meat), Australia (47.64 kg/capita/y, 77.7% bovine meat), Chad (45.42 kg/capita/y, 60.6% bovine meat) and Zimbabwe (44.16 kg/capita/y, 95.8% bovine meat). It must be noted that Australia increased its red meat consumption by 12 kg/capita/y in 2020 compared to 2019. In other countries, such as the Democratic Republic of Congo, Mozambique and Liberia, the consumption of red meat was below 1 kg/capita/year. This consumption is related to the animal production in each country and to the income. The change in consumption in 2020 compared to 2010 is presented in Figure 1b. It is possible to see that the majority of countries have reduced red meat consumption, this reduction being greater than 10 kg/capita/y in the Bahamas, Uruguay, New Zealand, Mali and Greece. With regard to Uruguay, this difference is essentially due to a major decrease (>7 kg/capita/y) in meat consumption in 2020 compared to 2019. In some other countries, there has been an increase in red meat consumption in the last 10 years, with an increase greater than 20 kg/capita/y in Mongolia and Tajikistan and above 10 kg/capita/y in Malta and Chad. Henchion [5] reported the trends in red meat consumption by region from 2000 to 2017 and, similar to the tendencies in the 2010–2020 period studied in the present work, red meat consumption decreased in all regions except for south-east Asia, although there were exceptions for some specific countries, both with increases and decreases in red meat consumption. Moreover, the same tendency has been predicted to continue between 2021 and 2030 in the OECD/FAO report [3], with an increase in beef consumption in China and a decrease in America, and a decrease in lamb consumption in Near Eastern and North African countries. 

The reduction in red meat consumption in some countries, such as Spain, Uruguay and Mexico, has also been reported in other works [9,10,11], in agreement with the data provided by FAOSTAT [8]. The main reasons for the reduction are variable and are related to the economic situation, sustainability or health concerns, as well as individual factors [12]. This red meat reduction is, in general, linked to an increase in poultry consumption (see Section 2.3.) and in some countries also an increase in pork consumption (see Section 2.2.)

### 2.2. Pork Consumption

**Figure 2:**
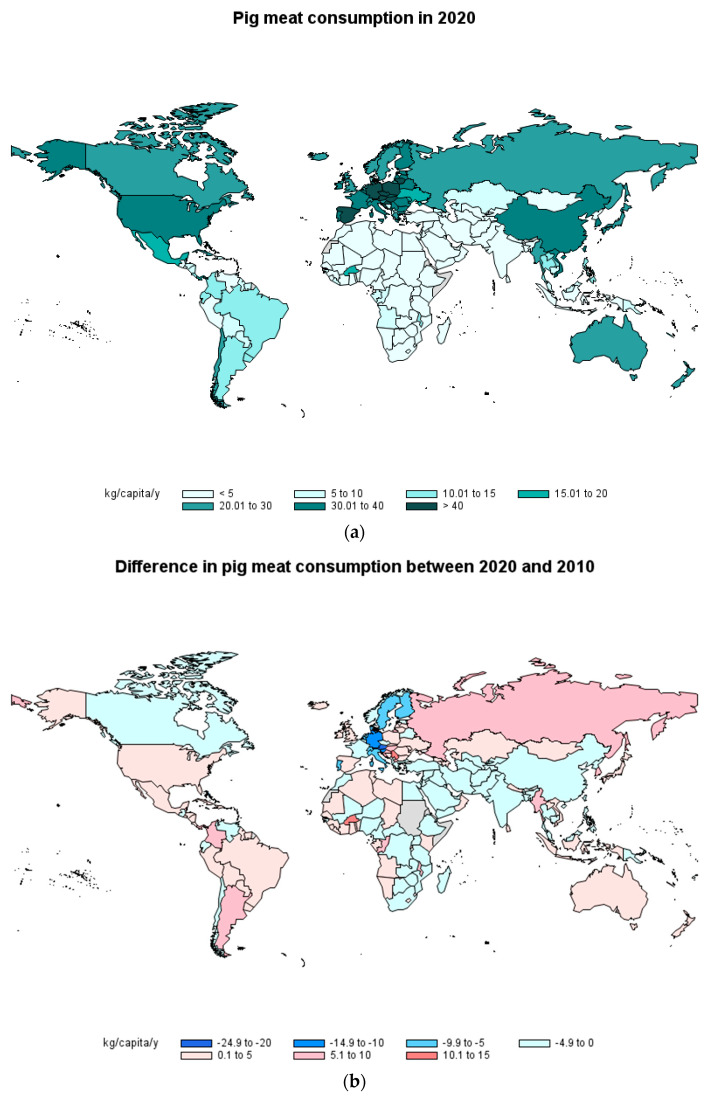
Consumption of pork in 2020 by country (**a**) and difference in consumption of pork between 2020 and 2010 (**b**) in kg/capita/year (Source: FAOSTAT [8]).

Regarding pork consumption, important differences between countries can also be found (Figure 2a). It is clear in the map that Muslim countries (and Israel), where the consumption of pork is not allowed due to religion issues, show a very low consumption per capita, together with countries known for having a dominantly vegetarian diet. There were 11 countries/regions with pork consumption higher that 40 kg/capita/year (4 with consumption higher than 50 kg/capita/year). Nine of these high pork consumer countries were European (Poland, Spain, Lithuania, Croatia, Hungary, Austria, Czechia, German, and Montenegro) and the other two regions were Hong Kong and Macao, both of these being in China. The most important decrease in pork consumption between 2019 and 2020 was in Denmark (10 kg/capita/y). A recent study carried out in Lithuania reports that, according to the FAO data, pork consumption in this country is very high and has been maintained over the last 7 years [13].

Compared to 2010, in 2020, pork consumption had decreased in some countries, e.g., several European countries and Canada and China, and has increased in others, e.g., Russia, United States, Australia and New Zealand, most of the Central and South American countries and some African countries (Figure 2b). For example, Serbia and Burkina Faso have increased their pork consumption by more than 10 kg/capita/y and, on the other side, Austria, has decreased consumption by more than 20 kg/capita/y, and Slovenia and Germany by more than 10 kg/capita/y. In Japan and Uruguay, pork consumption has also increased, according to some recent studies [9,14]. 

Trends in pork consumption between 2021 and 2030 are expected to follow along the same lines as seen between 2010 and 2020 given that, according to OECD/FAO [3], it is estimated that pork consumption will decrease in Europe and in most developed countries, and increase in Latin America and several Asian countries.

### 2.3. Poultry Meat Consumption

**Figure 3:**
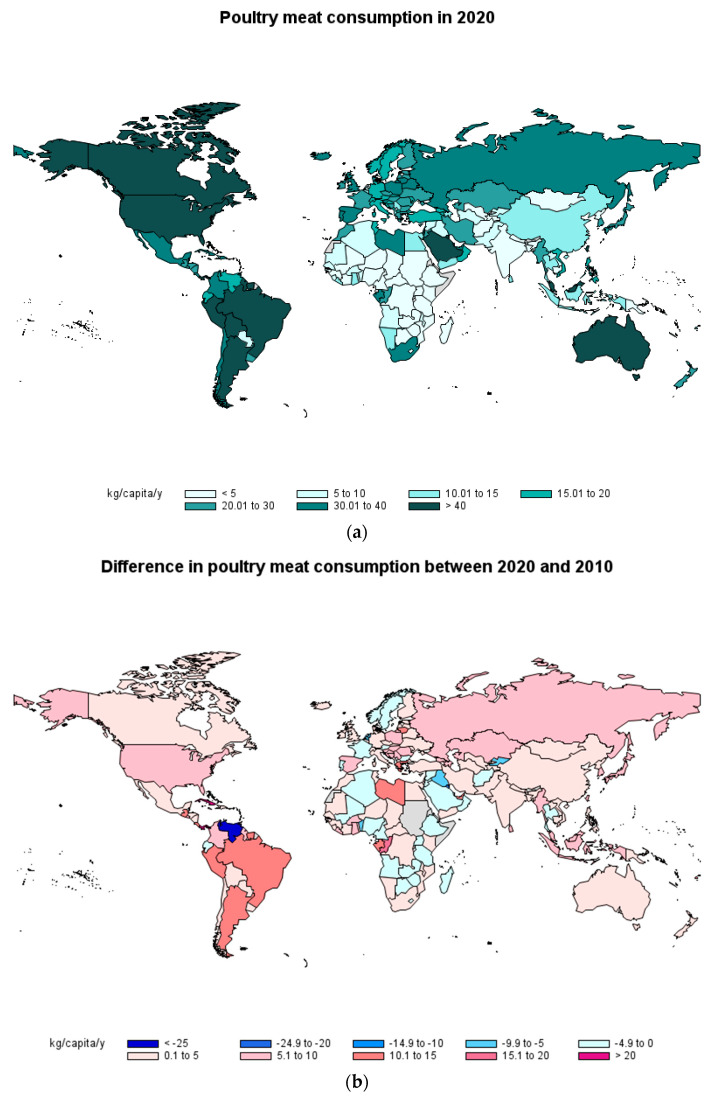
Consumption of poultry meat in 2020 by country (**a**) and difference in consumption of poultry meat between 2020 and 2010 (**b**) in kg/capita/year (Source: FAOSTAT [8]).

Poultry meat consumption in 2020 (Figure 3a) was higher than 70 kg/capita/y in 1 country (Saint Vincent and the Grenadines), higher than 60 kg/capita/y in 4 countries (Israel, Samoa, Trinidad and Tobago and Antigua and Barbuda), higher than 50 kg/capita/y in 7 countries/regions (United States, Saint Lucia, Hong Kong (China), Panama, Bahamas, Jamaica and Malaysia), and higher than 40 kg/capita/year in 16 countries. It has to be noted that, in Saint Lucia as well as in Comoros, poultry consumption in 2020 was 25 kg/capita/y higher than in 2019, and was 11 kg/capita/y higher in Macao (China). On the other hand, poultry consumption was reduced by 10 kg/capita/y in 2020 compared to 2019 in Fiji. Despite this, poultry is the most widely consumed meat in many countries. However, several African and South-East Asian countries consumed less than 5 kg/capita/y, either due to a food security issue or because they are countries with a vegetarian diet.

The change in poultry meat consumption between 2010 and 2020 (Figure 3b) shows that Venezuela has reduced its poultry consumption by more than 24 kg/capita/y and Kuwait by more than 20 kg/capita/year. Saint Kitts and Nevis reduced poultry consumption by more than 20 kg/capita/year. On the other hand, Panama has increased poultry consumption by more than 25 kg/capita/year and Cuba by more than 20 kg/capita/year. Sixteen countries have also increased poultry consumption by more than 10 kg/capita/year. When pork and poultry meat was considered together with white meat, the evolution by region showed an increase across all regions (between 2010 and 2017) except for the Eastern Mediterranean Region [5], which is broadly similar to what is seen when individual countries are studied.

According to the OECD/FAO report [3] this trend in increasing poultry meat consumption is expected to continue for the coming years, accounting for 52% of the additional meat consumed between 2021 and 2030 and being a major source of meat in the diet of a large part of the population.

In contrast with red meat consumption, most countries have increased poultry meat consumption in 2020 compared to 2010. This is probably related to its lower prices, product consistency and adaptability and more favorable protein–fat ratio [3], and it is an indicator of the change in meat consumption habits by consumers, whether due to economic or to health issues.

### 2.4. Main Concerns and Motivations for Changes in Meat Consumption Patterns

Consumer demand for meat and meat product characteristics has changed over the period and depends on certain intrinsic and extrinsic factors, either psychological, sensorial or marketing [12]. The characteristics that consumers demand of meat vary between countries and within countries [9,10,14,15]. Recently, some extrinsic properties, such as welfare-friendly, sustainable and healthily produced meat, are becoming more and more important. 

In recent years there has been a campaign against red meat and meat product consumption [16], with claims about the health problems associated with consuming it to excess, as well as the environmental impact and animal welfare aspects of its production [17]. These, together with economic aspects such as the increase in the price of red meat or the decrease in consumer incomes, are probably the reasons for the reduction in red meat consumption and the increase in poultry meat consumption [18,19,20] and, in some cases, also in pork consumption [9,11]. In fact, a study carried out in Uruguay reported that the decrease in meat consumption there was mainly due to three reasons: price, which was the most important with 46% of responses, health issues with 21%, and dietary changes with 19% of responses [9]. Furthermore, the increase in beef prices in Mexico has been reported to be the most important cause of the decrease in beef consumption observed in this country and the consequent increase in consumption of the more affordable types of meat such as chicken and, recently, pork [11]. Indeed, meat consumption is higher for those consumers from high or medium high socio-economic classes than for those of a low socio-economic class [10]. Moreover, religious and cultural issues could also limit meat consumption in some countries such as Ghana and India [21,22]. In addition, to overcome what is known as the meat paradox [23], i.e., “I like animals, but at the same time, I eat animals”, consumers have implemented different strategies [7], one of which is to reduce or stop meat consumption even though another strategy is to continue eating meat while avoiding the unpleasant issues.

In addition to the fact that it is an important source of nutrients [1,2], meat consumption is a cultural issue and this is one of the main reasons it is eaten in many countries [7,9,24]. Meat is part of gastronomic traditions, religious celebrations, local fairs or family events [25,26] and it is seen as a factor in socializing and identity [21]. Accordingly, motivations for meat consumption have also been reported to be bodily characteristics, the pleasure of eating meat, its nutritional properties and healthiness [10,14,15,21]. Meat consumption has also been considered natural, normal, necessary and nice, according to the 4N theory [27]. Thus, it could be very difficult to change these traditions, habits and beliefs [7], although they are evolving and changes are taking place more or less rapidly as can be seen in previous sections. Therefore, it is very difficult to know what role meat will have in the society of the future [7]. As an example, in non-meat-eating countries such as India, meat consumption has increased in recent years, although this consumption would be mainly related to income, dietary habits and cultural issues [22]. 

### 2.5. Main Types of Changes in Meat Consumption Patterns

These motivations for and barriers to meat consumption, mainly due to nutritional, environmental, social and ethical considerations, might drive changes in meat consumption patterns. As has been showed in Section 2, one change is switching the type of species the meat comes from, i.e., a reduced consumption of red meat and increased consumption of poultry. With this, consumers expect to reduce the risk of the health problems associated with red meat consumption, red meat having been considered an ‘unhealthy food’ by the EAT-Lancet report [16], although the way this risk is calculated has been criticized and the risks of reducing meat consumption have been explained [1,28].

This could also be applied to another change in meat consumption patterns which is the reduction in meat consumption or its total avoidance, i.e., to the total or partial substitution of meat by meat alternatives and meat analogues. This is a growing tendency in many countries where these products are available in the supermarkets. Some of these products have been always in the human diet and others, more processed products and analogues, are newer.

Moreover, motivations for and barriers to changes in meat consumption might also support more sustainable practices in agriculture and animal husbandry. This will shift consumption patterns towards more sustainable or environmentally friendly products, as well as towards products that are animal welfare friendly. An increased consumption of offal would also drive the growth of the circular economy. However, it must be noted that not all strategies that are good for sustainability are good for animal welfare and vice versa [29].

## 3. Consumption of Meat Produced in a Sustainable and Organic Way

As explained above, an alternative to conventional meat consumption when there are concerns for sustainability and animal welfare is the consumption of meat produced more sustainably and/or following animal welfare standards. It is worth noting that the environmental impact of meat production is difficult to determine as it depends on a multitude of factors such as production systems, the competition for resources useful in other food or feed production or the metric used for its calculation [30,31].

**Figure 4:**
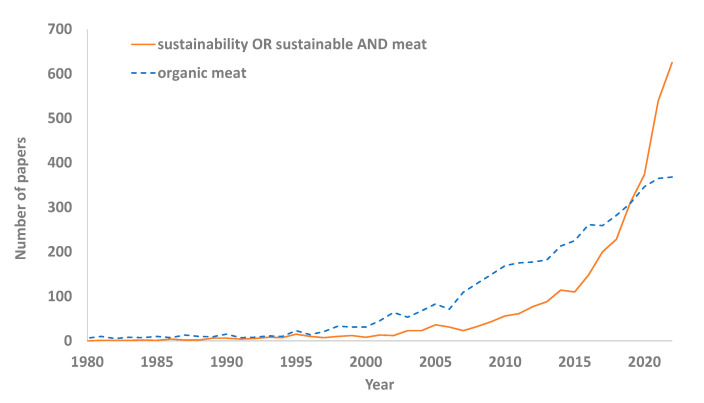
Number of papers referenced in PubMed that included each term from 1980 to 2022.

The number of scientific publications that include the term sustainability or sustainable meat have increased in recent years. Within sustainability, studies considering organic meat are also very important (Figure 4). This indicates the importance of this aspect within the scientific community and society in general.

### 3.1. Consumption of Sustainable Meat

Most consumers are aware of sustainability but, in general, they associate sustainability with the environment, and they are not aware of the broader meaning of this concept, which also includes economic viability and social equity aspects [32,33,34] and may also include temporal and development dimensions [35]. Although sustainability has been stated as a reason for decreasing meat consumption, in general, its impact on consumption is low [36,37] and country-dependent [38], probably due to the socio-cultural role of meat, the pleasure gained from consuming it [7], a strong attachment to meat [39] or the low or lack of awareness of the impact of meat production on sustainability [19,40]. Additionally, in some countries such as Japan and India, where meat consumption is very low due to cultural reasons, it would hardly be necessary to reduce its consumption due to environmental concerns [14]. 

It should be noted that one action in trying to deal with the concerns about ethical and sustainability issues is to increase the consumption of products produced in a more sustainable way. Furthermore, consumers considered sustainable foods safer and of higher quality [32]. One handicap of these products is that they have or are perceived to have a higher price than the conventional products. Thus, it is important to understand the willingness of consumers to pay for them. There are several scientific papers that consider this aspect, although they do not cover all the regions nor all the foods. In this regard, a meta-analysis carried out in 2021 [41] considered 80 papers, 43% from Europe, 26% from Asia, 26% from North America and 5% from Oceania. Sustainability and willingness to pay for sustainable food is probably an issue in some regions of the world, but, in other regions, sustainability is not an issue, perhaps because other aspects such as food security might be more important for consumers. This meta-analysis [41] showed that the willingness to pay for sustainable food increases when income increases and in middle-aged consumers (between 31 and 55 years old). It also showed a higher willingness to pay for these products in Europe and Asia than in North America and Oceania. Moreover, the willingness to pay for organic meat (39%) was reported higher than that for seafood (17%) and drinks (25%), and lower than that for dairy products (35%) and fruit and vegetables (39%). However, another study showed that the willingness to pay, studied as purchasing power parity, was higher for meat and dairy products than seafood, nuts, vegetables and fruit [42]. This is probably due to the greater importance that is normally given to environmental and animal welfare issues in meat and other animal products compared to other foodstuffs. Moreover, this study also concluded that marketing strategies for sustainable products are important, as the willingness to pay is higher for eco-labelled foods and, among these, organic-labelled products.

### 3.2. Organic Meat Consumption

One particular case of sustainable food is organic food, which is considered sustainable and healthier by most consumers [43,44]. In addition, some organic foods have been on the market for a long time and the labels and the concept are familiar to consumers [42]. Moreover, according to the EU legislation [45], organic production delivers “*publicly available goods that contribute to the protection of the environment and animal welfare, as well as to rural development*”. However, several aspects need to be considered when sustainability between organic and conventional production systems are compared and the best performance does not always flow in the same direction [46]. 

A meta-analysis that studied the importance of sustainable attributes in the willingness to pay for a product showed that consumers had the highest willingness to pay for organic products (38.1%), followed by fair-trade (30.5%), animal welfare (29.5%), environmentally friendly (21.3%) and locally produced (21.1%) [41]. Similarly, organic labels seem to be more valued by consumers than environmental sustainability labels [42]. Moreover, the review carried out by Hemmerling [47] states that protecting human health was the most important factor for purchasing organic meat, followed by far by taste, environmental protection, lack of pesticides and safety. However, it is important to bear in mind that, as reviewed by Bourn and Prescott [48], other aspects such as high price, low availability, unsatisfactory quality, satisfaction with current food purchases and unfamiliarity with ‘organic’ issues would be reasons for not purchasing organic foods.

The nutritional aspects related to the fatty acid profile of organic vs. conventional meat are highly connected to the diet the animals follow. In general, grass-fed ruminants have a fatty acid composition more desirable from a nutritional point of view than grain-fed animals, with higher polyunsaturated fatty acids, omega 3, EPA (eicosapentaenoic acid), DHA (docosahexaenoid acid) and CLA (conjugated linoleic acid), but lower or similar monounsaturated fatty acids [49,50,51]. Probably due to this, a meta-analysis showed that organic meat has higher polyunsaturated fatty acids, omega 3 and omega 6, and lower monounsaturated fatty acids than conventional meat, although these differences depend on the species from which the meat comes [52]. Grass-fed beef has been reported to be more nutritionally desirable than organically produced beef and, in the same vein, a recent review [49] concluded that human health benefits, in terms of fatty acid content, are superior when grass-fed beef is consumed, both organically and conventionally produced.

From a sensory point of view, a similar situation might probably apply since, if organic is grass-fed and conventional is grain- or concentrate-fed, differences can be important. However, it is not clear if these differences would be so important if organic and conventional meat were both grass-fed or grain-fed. Moreover, it is probable that other management and husbandry factors, together with breed or genotype, might also influence the sensory characteristics of the meat [53]. Consumers tend to expect organic meat to be better from the sensorial point of view than conventional meat. However, a Danish study showed that when consumers taste organic pork compared to conventional pork, organic pork was scored lower in terms of taste, juiciness and overall acceptability, with no differences in tenderness, even though expectations were higher [54]. However, other studies reported that the tenderness of organic pork could be lower due to the physical activity and slower growth rate of the animals compared to intensively reared pork [55].

Therefore, the higher quality of organic meat, when compared with conventional meat, is more related to ethical, animal welfare and sustainability qualities than it is to nutritional or sensory qualities. Additionally, as well as other external or personal factors such as lifestyle or personal experience, information provided to consumers might influence their expectations of and sensory preference for organic meat [54], and these are important aspects to be taken into account in order to reach and satisfy consumer demands.

### 3.3. Offal Consumption 

Edible offal or edible by-products are rich in protein and essential amino acids, vitamins and minerals and they can be used directly or by applying valorization strategies, either as food, as a food ingredient or as other types of product (e.g., pharmaceutical, biomedical or feed products) [56,57,58]. Therefore, the use of edible by-products could be a solution to animal protein demand, and also to increase sustainability due to the reduction in waste from slaughterhouses [59,60]. In fact, edible offal could be a source of nutrients in malnourished or low-income households [61]. Moreover, some edible by-products are part of traditional dishes in some regions and countries, e.g., haggis in Scotland, ‘callos’ in Spain, etc., but their consumption has generally decreased in most countries due to a number of reasons, as detailed by Llauger et al. [60]. 

**Figure 5:**
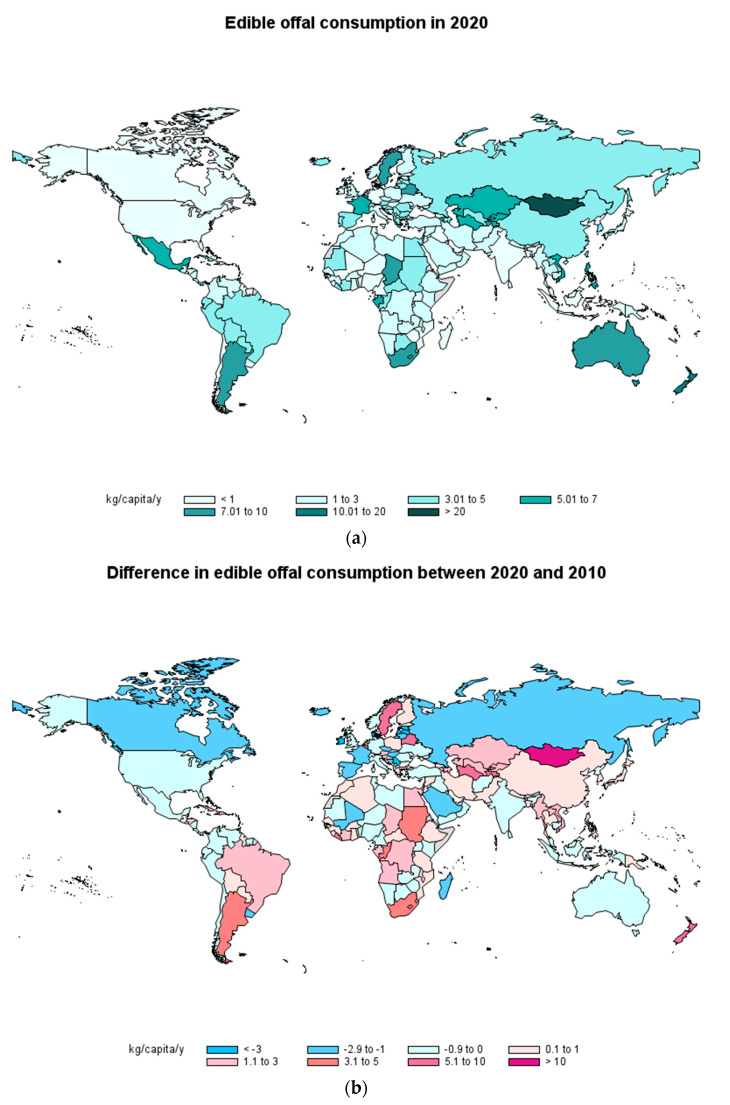
Consumption of edible offal in 2020 by country (**a**) and difference in consumption of edible offal between 2020 and 2010 (**b**) in kg/capita/year (Source: FAOSTAT [8]).

Figure 5a shows the consumption of edible offal in 2020 by country according to FAOSTAT [8]. The most important consumption was in Central and South America, Asia and Oceania, as well as in some countries of Europe and Africa. Thus, the highest values were found in Hong Kong (China) and Mongolia (52.7 and 40.7 kg/capita/y, respectively), followed by Saint Lucia (25.9 kg/capita/y) and Belgium (10.7 kg/capita/y). Argentina and Australia also have an offal consumption higher than 9 kg/capita/y. The lowest consumption (<0.5 kg/capita/y) was reported in North America and in some African and European countries. Thus, Canada, Denmark, India, Iraq, Madagascar, the Maldives, Mozambique, Sao Tome and Principe, the Solomon Islands, Sri Lanka and the United States of America were the countries with the lowest consumption.

With respect to the changes in edible offal consumption between 2020 and 2010 (Figure 5b) different patterns can be seen. Some countries that have decreased their consumption by more than 3 kg/capita/year were Ireland, Denmark, Serbia, Djibouti, Estonia, Luxemburg and Latvia. In contrast, 13 countries have increased edible offal consumption between 3 and 10 kg/capita/y, and 3 more (Hong Kong (China), Mongolia and Saint Lucia) have increased it by more than 20 kg/capita/y. Oh and See [62] reported that Chinese consumers preferred pork offal in comparison to Japanese consumers who preferred both, as well as loin and Boston butt. However, this study was published in 2012 and it would be worthwhile to see if the same pattern is still valid today. Regarding types of cattle and goat offal, in Kumasi (Ghana), stomach (53%) and liver (38%) were the most preferred offal over kidney, intestine, lungs, heart and tongue, mainly because of their nutritional value, delicacy and price [63].

Motives for the consumption of offal or products containing offal could be familiarity and previous experiences with these products, sensory perception of the products and environmental and sustainability issues. Some of these reasons can also be barriers. In fact, it has been reported that barriers for offal consumption were mainly related to the taste and visual appearance of raw offal, the association of these organs with the live animal, not being accustomed to its consumption because they did not consume it during their childhood, health and safety issues, food neophobia and a lack of knowledge of the benefits of its consumption [59]. These barriers could probably be broken down or reduced by the use of by-products as ingredients in other foods [59,60]. In this regard, reasons for the consumption of offal-derived products could be related to health benefits, to past experience, the consideration of offal as a natural product, and using offal product as a seasoning while always ensuring the product is safe. The barriers would be the same as for ‘raw’ offal plus the processing issues [59]. However, some segments of consumers can be found who have a different opinion regarding this consumption, e.g., pro-offal-based meat products, health and environmental consciousness and reluctance to consume offal-based meat products [60]. In Turkey, meals containing offal are very common. Thus, a recent study aiming to evaluate the opinion of tourists towards typical Turkish meals containing offal was carried out and it showed that most tourists have neophobia toward new foods as they declared themselves to be afraid or hesitant about eating new foods, to not trusting new and different foods, not consuming foods they did not know and, moreover, to being very picky about the food they eat [64].

## 4. Meat Alternatives Consumption

Due to the increasing concerns about the ethical and environmental aspects of meat production, there has been an increase in the production and consumption of meat alternatives, although the ‘advantages’ are not always clear. Consuming meat alternatives is another way of reducing the consumption of animal food products, specifically meat. Even though it is possible to find these meat alternatives in some markets [10,18,65], in others it can be more difficult, and availability is an important external factor that affects the consumption of these products [66].

**Figure 6:**
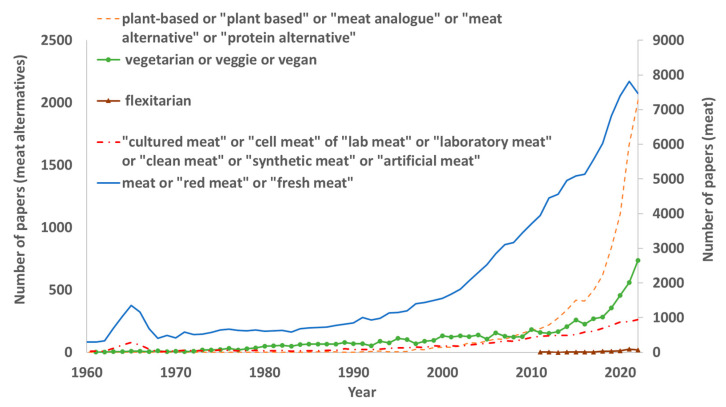
Number of papers references in PubMed that included each term from 1960 to 2022.

Moreover, the number of research studies including the terms plant-based, meat alternative, protein alternative or meat analogue has increased considerably in the last decades (Figure 6), indicating the important investment in this type of alternative [67]. In addition, but to a lesser degree, research into vegetarian and vegan issues has increased, as well as research into cultured ‘meat’ and into flexitarians to a lesser extent. In fact, the high interest in meat alternatives is probably related to the increase in flexitarian and mainly vegetarian and vegan consumers.

### 4.1. Types of Meat Alternatives 

Meat alternatives can be plant-based (including pulses), fungus-based and algae-based products (hereafter collectively referred to as PAF-based). They can also be insect-based and cultured or lab-grown ‘meat’. In most cases they can also be considered as meat analogues because they try to mimic meat, mainly in terms of their visual appearance (e.g., burgers and sausages) and in-mouth sensory characteristics (e.g., flavor, odor and texture). Consumption of these alternatives is different and depends on a several factors and motivations. In this section the concept and availability of these alternatives has been reviewed.

PAF-based alternatives are products made with plant, fungus or algae products that can be eaten in their natural state or after processing, either low-processed (first generation) or highly processed products (second generation) [36]. Natural PAF-based products have formed part of human meals for centuries, not only for vegetarian and vegan consumers. However, the type of product depends on the culture and customs. For instance, algae products are common in Asian diets, either fresh or dried [68], while in Western countries these are not commonly eaten. Similarly, first generation plant-based products, such as tofu and seitan, have been part of Asian diets for years and were less common in other countries [36], or only consumed mainly by vegetarian and vegan consumers. Second generation plant-based products are intended to enhance the sensory experience and are therefore generally more processed [36] and they are more or less available on the markets [7,18].

Another important source of protein is insects. For many years, insects have been part of the human diet in some countries, due to cultural and religious practices. However, in other countries, the consumption of insects is viewed with disgust and as a primitive behavior [69] and, because of this, in these countries insects are one of the least accepted meat alternatives [70]. In other countries, such as Mexico, insect consumption is an accepted habit, although they are not consumed regularly, only in specific situations [24]. Insects can be consumed as a whole insect, either entire or after removing some parts, as ground insects or a paste added to another product or, finally, as an extract added to other foods, even though the extraction process could be very expensive [69]. 

Finally, another meat alternative is cultured ‘meat’, also known as cell ‘meat’ or lab-grown ‘meat’ or clean ‘meat’, even though the use of the term ‘meat’ might be criticized and creates confusion [7]. In this case, stem cells are selected, and then placed in a growth media and stimulated to allow cells to proliferate, differentiate and mature. After this they are scaled up and are then harvested and used to make products [71]. Currently, cultured ‘meat’ is only available on the Singaporean market, but will probably be approved soon in other countries.

### 4.2. Meat Analogues 

Some meat alternatives are meat analogues, since they try to mimic meat and the appearance and taste of meat products for a variety of reasons, such as the importance of what we see and perceive, the attachment of meat to human culture and social events, or the transition to a vegetarian diet [7,12,72,73]. However, this is not easy [74]. In fact, the lack of similarity with real meat products is one of the main reasons why they do not always satisfy consumers [75,76]. Meat analogues are produced by specific or new companies created for this purpose, but it has also been an opportunity for meat companies to adapt their meat processing facilities to produce a line of meat analogue products or to build new processing facilities for these products [10,18]. 

Consumers that like to consume meat analogues are consumers that would like to reduce their meat consumption either for health or other reasons, as well as flexitarian consumers who eat meat occasionally and consumers who like meat and do not want to give up its sensory qualities [18]. It is also reported to have been related to political ideas [77]. 

In any case, it is important that the consumer is not confused in the use of the term ‘meat’ in these products, because although meat analogues imitate meat, they are not meat and, consequently, might not have the same nutritional value of meat.

### 4.3. Motives for and Barriers to Meat Alternatives Consumption 

The acceptance of meat alternatives by consumers is influenced by psychological, marketing and sensorial factors [12]. For instance, cultural aspects and familiarity with the products and dietary habits, might partly explain these differences in acceptability [39,67]. Furthermore, the acceptability depends on the type of alternative, being lower for insects and cultured ‘meat’ than plant-based products and, of course, it also depends on the characteristics of the consumers, influenced by their familiarity with the product, attitudes and neophobia, among other reasons [70]. When a hybrid snack (made with meat and meat alternatives) was available as an alternative, this was preferred in more than half of the cases, followed by a lentil or bean snack, then by a seaweed snack and, as a last choice, by an insect snack [78]. It is therefore important to consider these meat alternatives as part of diets in order to reduce meat consumption, when such consumption is excessive, and not necessarily to eliminate meat consumption completely. 

It is important to take into account that, the information provided to the consumer is also very relevant and greatly influences the consumer’s acceptance of the product [79]. Due to this, it is important to keep consumers well informed, with reliable and trustful information [7] in order to avoid them being influenced by fake and highly sensationalist news. 

The motives for meat alternative consumption have been classified into product-related factors (i.e., food motivations such as taste, convenience, environment, healthiness and familiarity), psychological factors (i.e., attitudes and neophobia, disgust and related feelings) and external attributes (i.e., trust, social environment and cultural appropiateness) [70]. In fact, the narrative used by the producers and promoters of these products is that they are healthier, good for food security, good for animals and the environment, have better control in the production and taste like animal products [80]. However, not all of these points are precise enough and depend on a variety of factors or conditions. 

Concern for the environment is an important reason for the consumption of meat alternatives since PAF-based meat substitutes might be very important in reducing the environmental impact [39]. However, some of these points are not so clear-cut and depend on a number of factors or circumstances. For instance, depending on the process used to extract proteins from plants (e.g., wet or dry separation), the extraction could be more or less sustainable due to its requirements of energy, water and other compounds [81]. Moreover, depending on the metric used to calculate the environmental impact, and what is included in its calculation, cultured ‘meat’ could be more or less sustainable [31]. In any case, it is important to note that, even though reducing meat consumption and consuming meat alternatives could reduce greenhouse gas emissions and improve the environment, other actions such as changing some aspects of the way we live can improve the environment much more [73].

There are important differences in the protein content and nutritional characteristics of the different meat alternatives [82]. Even though some consumers might perceive the consumption of these alternatives to be better than meat consumption due to the aspects previously stated, there is not always evidence of these advantages. For instance, a beef patty has more fat, saturated fat and cholesterol than tofu but, at the same time, tofu has more sodium and less protein, iron and zinc than a beef patty [83]. Moreover, generally, the number of ingredients in meat alternative products and the risk of including some allergens is higher. In this regard, Ngapo [18] compared the number and type of ingredients in meat products and meat analogues, showing these differences. Similarly, an Australian study also reported differences in nutrients among meat alternatives available on the market; in some cases the alternatives were better than meat, but for other products or nutrients, they were worse [65].

The barriers to PAF-based meat alternative consumption have been reviewed by Jahn et al. [36], and were classified as structural barriers (i.e., limited availability, novelty and price) and motivational barriers (i.e., phobia of new products, socio cultural aspects, hedonic and health issues). Some of these barriers can also be motives for PAF-based meat alternative consumption, depending on the point of view from which one looks at them. For instance, sensory characteristics can be a barrier if the product is not attractive or tastes different from meat or a motive if the consumer dislikes the taste and appearance of meat. Another example is health, which can be a barrier if consumers consider meat nutrients very important, or a motive, if they consider meat not to be healthy due to (saturated) fat content. Moreover, specific concerns relating to potential food safety hazards in cell-based food have been reviewed by FAO and World Health Organization (WHO) [84] for the sourcing, production, harvesting and processing stages.

In addition, consumers do not necessarily consider meat substitutes to be more sustainable and healthier than meat, and this can be related to the fact that they are perceived as less natural [85], and acceptability is lower if perception of naturalness is lower [86]. Thus, meat substitutes have some challenges to meet, and they should provide some characteristics and advantages over meat in order to be considered positively by consumers. In this regard, Swedish consumers would switch to meat substitutes mainly if they carried labels with information regarding their advantages (antibiotics used, climate impact, animal care, etc.), if their taste was similar to meat and if their price was reduced [87].

## 5. Conclusions

Meat and meat alternative consumption depends on the country and region and also on the consumer’s attitude, behavior and personal situation. There is an evolution in consumption, but it is difficult to know what will happen in future years because of the tendencies towards more sustainable production and consumption, the economic situation, the pandemic situation and climate change that are already affecting arable and livestock production. Thus, it is very important to work towards producing meat, meat products and meat alternatives in a more sustainable way, and providing truthful and reliable information to consumers regarding the advances made and the advantages and disadvantages of eating the different products.

As an avenue for future research, it would be important to clarify the different aspects regarding the sustainability, nutrition, social and ethical issues of meat and meat alternatives because some of these are still not sufficiently clear. Moreover, because of the advances in data processing by means of deep learning and artificial intelligence tools, in the future it would worthwhile integrating all information related to the production and processing of meat and meat alternatives, i.e., all intrinsic and extrinsic factors, in order to try to have a global vision of the product and its characteristics and to allow the consumer to know the different features of the product.

## Data Availability

The data used in this work is available online as cited in the references.
